# Feasibility of Cobalt-Free Nanostructured WC Cutting Inserts for Machining of a TiC/Fe Composite

**DOI:** 10.3390/ma14123432

**Published:** 2021-06-21

**Authors:** Edwin Gevorkyan, Mirosław Rucki, Tadeusz Sałaciński, Zbigniew Siemiątkowski, Volodymyr Nerubatskyi, Wojciech Kucharczyk, Jarosław Chrzanowski, Yuriy Gutsalenko, Mirosław Nejman

**Affiliations:** 1 Faculty of Mechanical and Energy, Ukraine State University of Railway Transport, 7 Feuerbach Sq., 61050 Kharkiv, Ukraine; cermet-u@mail.com (E.G.); NeVlPa9@gmail.com (V.N.); 2 Faculty of Mechanical Engineering, Kazimierz Pulaski University of Technology and Humanities in Radom, Stasieckiego 54, 26-600 Radom, Poland; z.siemiatkowski@uthrad.pl (Z.S.); wojciech.kucharczyk@uthrad.pl (W.K.); 3 Institute of Manufacturing Technology, Faculty of Production Engineering, Warsaw University of Technology, ul. Narbutta 86, 02-524 Warszawa, Poland; tadeusz.salacinski@pw.edu.pl (T.S.); jaroslaw.chrzanowski@pw.edu.pl (J.C.); m.nejman@zaoios.pw.edu.pl (M.N.); 4 Department of Integrated Engineering Technologies, National Technical University Kharkov Polytechnic Institute, 2, Kyrpychova str., 61002 Kharkiv, Ukraine; gutsalenko@kpi.kharkov.ua

**Keywords:** WC, cutting tool, sintering, microstructure, durability, wear

## Abstract

The paper presents results of investigations on the binderless nanostructured tungsten carbide (WC) cutting tools fabrication and performance. The scientific novelty includes the description of some regularities of the powder consolidation under electric current and the subsequent possibility to utilize them for practical use in the fabrication of cutting tools. The sintering process of WC nanopowder was performed with the electroconsolidation method, which is a modification of spark plasma sintering (SPS). Its advantages include low temperatures and short sintering time which allows retaining nanosize grains of ca. 70 nm, close to the original particle size of the starting powder. In respect to the application of the cutting tools, pure WC nanostructure resulted in a smaller cutting edge radius providing a higher quality of TiC/Fe machined surface. In the range of cutting speeds, *v_c_* = 15–40 m/min the durability of the inserts was 75% of that achieved by cubic boron nitride ones, and more than two times better than that of WC-Co cutting tools. In additional tests of machining 13CrMo4 material at an elevated cutting speed of *v_c_* = 100 m/min, binderless nWC inserts worked almost three times longer than WC-Co composites.

## 1. Introduction

The wear resistance and durability of cutting tools are very essential factors for both producers of cutting tools and the industrial customer [1]. Tungsten carbide (WC) material is widely known for its applications as a tool material for machining and even rock drilling applications. The cutting inserts are generally manufactured through a powder metallurgy process followed by sintering, using the tungsten powder with a binding material such as cobalt, and usually, a heat treatment is added to enhance the life of the tool by reducing flank wear and cater to the wear of the tool [2].

Apart from its principal responsibility as the binder constituent, cobalt also provides the necessary toughness to the tool. So, by varying the amount of cobalt, the required hardness and toughness characteristics can be obtained [3]. Tungsten cobalt cemented carbide inserts have a wide range of applications, mainly for cutting cast iron, non-ferrous metals, and their alloys [4]. However, the binder itself is much softer than the cemented carbides, so that increase of the cobalt binder content weakens the overall mechanical properties of the cutting tool, and does not improve its wear resistance [5]. In addition, high temperature deformation and creep of tungsten carbide-metal alloys takes place, mostly due to softening of the metal binder and grain sliding at elevated temperatures [6]. To reduce this effect, sintered WC with an ultra-low cobalt (Co) content of 0.3, 0.6, and 1.0 wt.% was investigated [7]. Some graphite was added too in amounts of 0.3 and 0.5%. The extensive study performed by Vornberger et al. [8] demonstrated that the hardness of all WC-Co composite grades increases with decreasing Co content and decreasing WC grain size, but different combinations of Co content and WC grain size, retain hardness at elevated temperatures very differently.

To avoid lowered hardness as well as chemical resistance caused by the addition of metallic element, production of the single-phase polycrystalline WC has been investigated by the use of a variety of methods, such as pressureless sintering, hot pressing (HP), hot isostatic pressing (HIP), high pressure-high temperature technique (HPHT), and spark plasma sintering (SPS) [9]. Tsai investigated the effect of the process parameters on the mechanical properties of binderless pure tungsten carbide during a gas protection sintering [10]. At a sintering temperature of 1860 °C for 60 min, he obtained a mean particle size of 1.03 μm, and the resulting properties of the material were as follows: a relative density of 95.1%, a micro-hardness of 1718 kgf/mm^2^, and a fracture toughness of 5.97 MPa∙m^1/2^. However, sub-micron pure tungsten carbide (WC) compacts of about 200 nm grain size prepared by the HPHT in a condition of 1500 °C temperature and 5 GPa pressure had a much higher relative density of 99.2%, and fracture toughness of 8.9 MPa·m^1/2^ [11]. A nano-crystalline binderless tungsten carbide (nWC) was considered as a substrate for molds for precision glass molding [12].

A review of the binderless tungsten carbide [13] indicates that various methods have some advantages and shortcomings, and thus many efforts are undertaken to obtain better results at a lower expense. In our researches, the objective was to investigate peculiarities of the fabrication of cutting tool material based on WC without expensive additions, such as cobalt or nickel. As-prepared inserts open the possibilities to recycle tungsten carbide and reuse it for the fabrication of new cutting inserts. Another goal of no less importance was to obtain the proper tool material for cutting the difficult-to-machine abrasive materials of high hardness, which could replace in some applications very expensive superhard cutting tool materials, such as diamond or cubic boron nitride. Since the second objective is closely connected to the first one, our study consists of the analysis of the electroconsolidation process of binderless nWC, analysis of microstructure and properties of the sintered samples, and, finally, of cutting tests using the as-prepared inserts. In the present study, nanostructured pure WC cutting inserts were fabricated using the modified electroconsolidation method [14]. The method is advantageous because of substantial energy savings, a short sintering time of a few minutes, and reasonably low temperatures between 1300 and 1800 °C. In addition, fabrication of such cutting tools out of WC nanopowders does not require binders and eliminates expensive and time-consuming mixing procedures, which results in further savings. In this respect, researchers have provided a better understanding of the consolidation process and lead to a patented cutting tool WolCar material [15]. Further scientific benefits result from investigations of its structure, strength, wear resistance, and feasibility to the practical application for machining of the difficult-to-cut TiC/Fe composites. This material belongs to steel matrix reinforced composites that perform the highest elastic modulus and hardness among all machinable materials [16].

## 2. Materials and Methods

### 2.1. Preparation of Cutting Inserts

A starting material for the cutting inserts was WC powder delivered by Wolfram company (Austria). It was produced with the plasmochemical method, catalog number 74–0601, purity 99.95%, size of the particles was between 40 and 70 nm.

The cutting inserts were sintered using the electroconsolidation or hot-pressing method (FAST–Field Activated Sintering Technique) with a directly applied alternating current [14]. The device does not require additional units for pulse generation, its built-in transformer converts industrial AC, 50 Hz, to the high current of 5000–6000 A, which is the sole source of heating. During electroconsolidation, a pressing force is achievable up to 45 MPa, and temperatures up to 1800 °C. Dimensions of the fabricated specimens corresponded with standard inserts SNUN120408.

The as-sintered cutting inserts were sharpened using diamond abrasive disc 12A2-45 AC6 100/80 M1-01 (Poltava Diamond Factory, Poltava, Ukraine) at speed *v* = 25 m/s. The final geometry of the cutting edge was as follows:

- True Rake Angle γ = 0°;
- Side Frank Angle α = 6°;
- Side Cutting Edge Angle φ = 45°;
- End Cutting Edge Angle φ_1_ =45°;
- Cutting Edge Inclination Angle λ = 0°.

**Figure 1:**
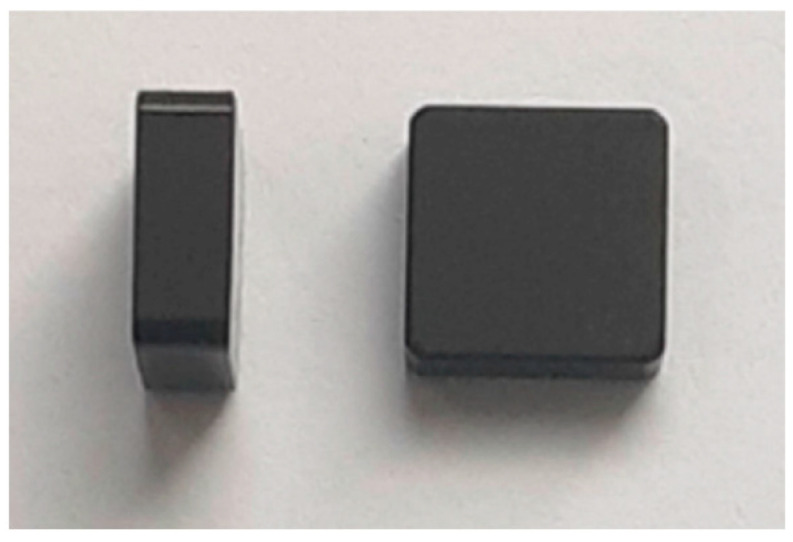
Photo of the nano-crystalline binderless tungsten carbide (nWC) cutting inserts.

Figure 1 presents the photo of sintered binderless WC cutting inserts.

### 2.2. Sintered Material Properties Investigations

As-obtained bulk material was investigated by means of X-ray diffractometry using the device DRON-4-07 (Bourevestnik, St. Petersburg, Russia) with copper Cu-Kα radiation. The Ni-selective absorbent β-filter was applied.

The microstructure of the sintered specimens was analyzed with a large specimen variable pressure scanning electron microscope (SEM) LEO1455 VP (ZEISS, Oberkochen, Germany). It allowed for magnification up to 900,000 times and resolution of 5.5 nm at 30kV in BSD–VP mode. Additionally, a field emission scanning electron microscope with Schottky electron emission source MIRA3 (TESCAN, Brno, Czech Republic) was used. It ensures low noise, resolution as high as 1.2 nm at 30 kV, and repeatability <1 μm in *x* and *y* axes. Additionally, the porosity of the sintered samples was determined through the relative density. The specimens were ground to obtain geometrical shape and the mass to volume ratio was compared to the standard WC density of 15.7 g/cm^3^.

**Figure 2:**
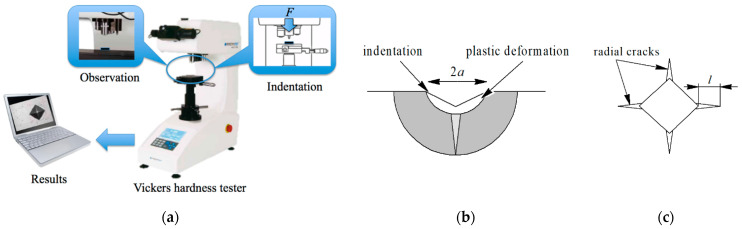
Measurement of hardness and fracture toughness: (**a**) Experimental stand; (**b**) Indentation in the brittle material; (**c**) Radial cracks.

Determination of microhardness and crack resistance of the obtained samples was carried out using a microhardness tester NEXUS 4504 (INNOVATEST, Maastricht, The Netherlands). Diamond Vickers indenter with angle α = 136° was applied under the load *F =* 10 kgf (98 N) for 10 s. From the measurement of the obtained indentation shown in Figure 2, the hardness value HV and fracture toughness *K_IC_* were calculated using Equations (1) and (2) [17].

(1) $$HV=\frac{kF}{{\left(2a\right)}^{2}},$$

where *k =* 1.854 is the factor dependent on the indenter form, *F* is the load [N], 2*a* is the average length of indentation diagonal shown in Figure 2 [μm].

Under the condition that the ratio *l/a* is 0.25 ≤ *l/a* ≤ 2.5, fracture toughness can be calculated as follows:

(2) $$K_{IC}=\zeta {\left(\frac{l}{a}\right)}^{-0.5}{\left(\frac{HV}{E\Phi }\right)}^{-0.4}\frac{HVa^{0.5}}{\Phi },$$

where *ζ* is the dimensionless constant (for ceramics *ζ =* 0.016), *E*–Young’s modulus [GPa], *l* is the crack length shown in Figure 2 [μm], Φ is the dimensionless constant (in this case Φ = 3).

### 2.3. Cutting Tests

For the cutting tests, a difficult-to-machine ceramic-particle-reinforced iron matrix composite was chosen. CPR-IMCs have been used in many fields due to their excellent performance [18], but they cause very intense wear of the cutting tools. The test was performed with a TiC/Fe composite, widely used for demanding wear-resistant components due to its significant advantages, such as low density, high hardness, and excellent chemical stability [19]. The principal microstructural features of this material are the TiC grain size and the nature of the iron binder phase as well as its distribution [20]. Its structure consists of an interconnecting interpenetrating network that contributes to enhanced engineering properties of the composite. The composition of machined samples was vol.% 45 TiC and vol.% 55 Fe (see Table 1). The experiments were performed at different cutting speeds from *v_c_* = 15 up to *v_c_* = 40 m/min and feed rate *f_n_* = 0.1 mm/rev. Cutting depth was *a_p_* = 0.2 mm. The machining parameters were calculated according to the recommendations of Sandvik Coromant, considering also the results of our own initial research. The tests were performed using the machine tool 16K20 type (Krasny Proletarii company, Moscow, Russia). An additional comparative cutting test was performed with 13CrMo4 steel at an increased cutting speed of *v_c_* = 100 m/min. The chemical composition of this material is shown in Table 1.

In the durability tests, the critical flank wear was considered *VB* = 0.4 mm. After reaching that point, the cutting edge was considered worn out. For a comparison, similar cutting tests were performed using standard WC cutting inserts with cobalt binder available in the market, denoted BK6 and BK8 with 6 and 8 wt.% of Co, respectively, produced by Kirovgrad hard alloys plant (KZTS, Kirovgrad, Russia). Additionally, cubic boron nitride (CBN) indexable inserts delivered by Walter company (Waukesha, WI, USA) were used in comparative tests.

## 3. Results

### 3.1. Phase Composition

**Figure 3:**
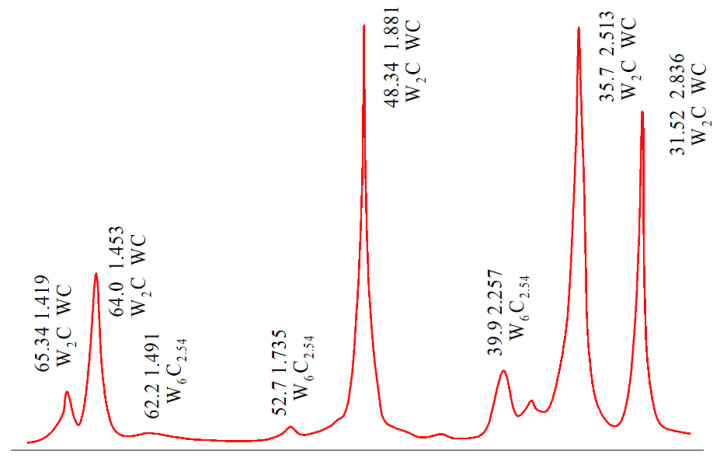
XRD of the WolCar obtained with electroconsolidation at *T* = 1700 °C and *P* = 45 MPa, for 2 min.

Figure 3 shows the results of X-ray diffraction phase analysis (XRD). XRD revealed that apart from the WC phase, the material contains brittle phase W_2_C and non-stoichiometric tungsten carbide W_6_C_2.54_. When sintering pure WC powder, a small amount of tungsten hemicarbide W_2_C is unavoidably formed because the surface of the WC powder becomes oxidized when the fine powder is handled in the air. It should be expected that the presence of W_2_C in the bulk sintered WC material might reduce its mechanical properties [22].

### 3.2. Microstructure

**Figure 4:**
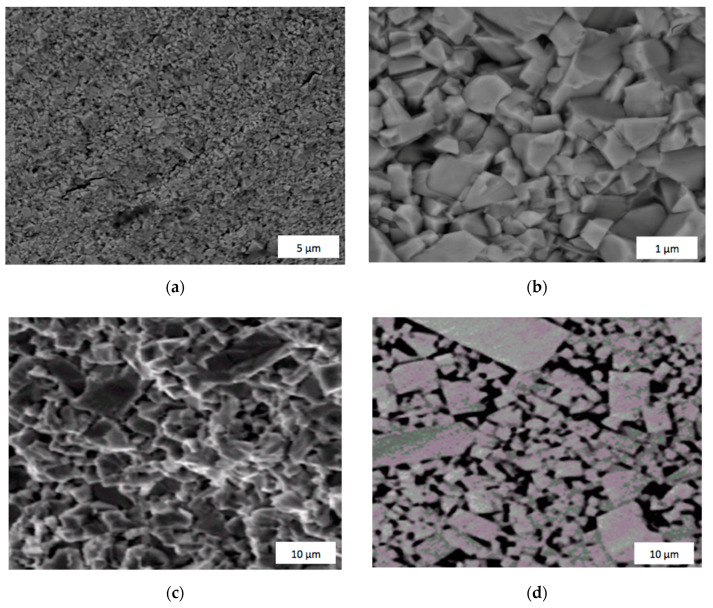
The fracture surfaces of sintered tungsten carbide (WC): (**a**) and (**b**) pure WC (WolCar) using the electroconsolidation method at *T =* 1700 °C and *P* = 35 MPa during 3 min; (**c**) Standard blend of 8 wt.% Co and 92 wt.% of WC using conventional vacuum sintering method at *T =* 1350 °C for 1 h, the surface without etching; (**d**) The same as (**c**), but after etching.

The results confirmed the main advantage of elctrocolsolidation, where reduced temperature and shorter sintering time can prevent grains from growth, ensuring enhanced mechanical properties of the bulk material. Figure 4 shows examples of the fracture microstructure of WolCar after electroconsolidation and sintered WC-Co composite. In the case of the WolCar sample, some grain sizes are retained close to the dimensions of the starting powder particles of ca. 70 nm. The grains and pores in the composite with 8 wt.% Co binder are of micron size, as is seen from Figure 4.

During electroconsolidation of WC nanopowders, it was found that the pressure should be applied to the sintered sample only when the temperature reaches 1000 °C, to allow the air to leave the sample body and thus to avoid pores formation. Moreover, large isolated pores may appear at high heating rates due to unsteady densification in different areas of the sintered volume. Especially, a high probability of pore formation occurs at heating rates close to 500 °C/min, which is the best rate from the perspective of successful consolidation of the dense material. Thus, to eliminate this undesirable phenomenon, the heating rate should be differentiated and precisely controlled. It was found that the pores do not appear when the heating is performed in the following stages:

- First, before the temperature *T_1_* = 300 °C is reached, the heating rate is kept at *h_r1_* = 50 °C/min;

- Next, the heating rate is increased up to *h_r2_* = 250 °C/min which is kept until the temperature *T_2_* = 900 °C is reached;
- Then the temperature is kept unchanged at the level *T_2_* = 900 °C for 2–3 min (densification process);
- Finally, the temperature is increased up to *T_3_* = 1700 °C with a heating rate of *h_r3_* = 500 °C/min (sintering process).

This process with differentiated heating rates ensures that all residual CO gas leaves the sample allowing for a structure without pores to be obtained.

### 3.3. Mechanical Properties

Mechanical properties of the sintered samples are shown in Table 2. For comparison, the results for three samples from sub-micron WC powders are provided, as well as the ones for standard WC-Co material with 92 wt.% of WC and 8 wt.% of Co binder, available on the market under the designation BK8.

It can be seen from Table 2, that the submicron grain sizes were retained after submicron starting powder was sintered at 1630 °C (sample #1) and for WolCar sintered at 1750 °C. However, the WolCar sample exhibited much higher density, obtainable for submicron powders after a much longer time but it was accompanied by considerable grain growth.

From the perspective of the cutting tools application, fracture toughness and hardness are of crucial importance. For the samples from submicron starting powders, both HV10 and *K_IC_* reduced along with increased grain size. The sintered WolCar samples exhibited fracture toughness close to sample #1 but with much higher hardness. Even though its *K_IC_* was smaller than that of BK8, the hardness was more than 60% higher.

### 3.4. Cutting Tests

**Figure 5:**
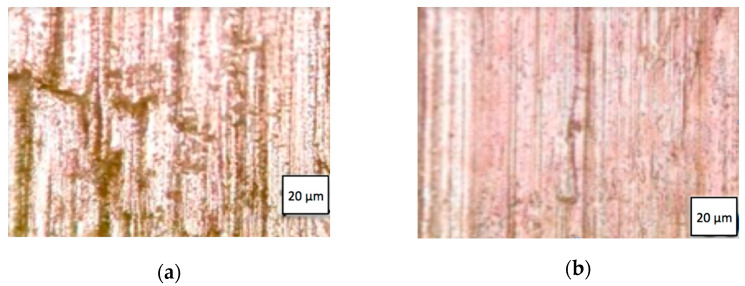
Quality of the TiC/Fe composite surface machined with different inserts: (**a**) BK6; (**b**) WolCar.

The WolCar inserts prepared as described in Section 2.1, had a substantially smaller radius of the cutting edge than that of typical WC-Co inserts available in the market. The cutting edge radius of the WolCar insert was 12 μm, while in the typical edge of the WC-Co insert BK6 (with 6 wt.%Co) it is 28 μm [23]. It is widely recognized that the cutting tool geometry has an effect primarily on surface roughness [24], but in addition, some authors indicate its influence on the intensity and course of dynamic load [25]. In our study, the machined surface of the TiC/Fe composite after cutting with WolCar inserts exhibited much higher quality and surface integrity than that after BK6 inserts. The difference is distinguishable visually, which is illustrated in Figure 5.

In terms of surface integrity, prows, microvoids, and microcracks can be identified in the surface layer [26] of the BK6 machined TiC/Fe composite. Such a surface requires further finishing, e.g., the grinding process. In contrast, the roughness *Ra* = 1.2 μm achieved after cutting with WolCar insert corresponds with that achieved by grinding, which is usually between *Ra* 0.1 μm and 2 μm [27]. In fact, the surface machined with WolCar insert exhibited the quality of a ground one, and it was also free from burned areas that typically appear during grinding.

In the dry cutting conditions, a built-up edge formation was notified on the WolCar cutting edges when the cutting speed increased up to *v_c_* = 40 m/min and more. However, the dimensions of the phenomenon were rather small and the application of a lubricant would overcome the issue.

An additional comparative cutting test was performed with 13CrMo4 steel at an increased cutting speed of *v_c_* = 100 m/min. In such harsh conditions, when the temperature in the cutting area might have reached 1000 °C, the BK6 edges became worn out after 10–12 min, while the WolCar inserts were able to work as long as ca. 30 min. It is most likely that the BK6 material lost its wear resistance at elevated temperatures due to the cobalt presence, while the abrasive wear resistance and high hardness of binderless WolCar were retained.

## 4. Discussion

In the case of typical difficult-to-cut materials, keeping high machined surface quality and improving machining efficiency has always been a key issue [28]. Considerable efforts have been made to improve the cutting performance of carbide tools in conditions without cooling and lubricating fluids and at elevated cutting speeds, when the carbide tool is subjected to more severe friction and wear, leading to the reduction of service life [29]. Accordingly, fracture toughness and hardness are the key characteristics of cutting tools.

An increase in the fracture toughness of nanostructural WC material compared to the known WC materials sintered out of submicron powders can be attributed primarily to the high dispersion of the grains and strong bonds between them. These characteristics were achieved through a short time of the sintering process and the formation of the contact necks between the neighboring grains.

It should be noted that the sintering mechanism of the tungsten carbide nanopowders is not known in full, even though there are reports on promising results [30,31,32]. In the case of the proposed electroconsolidation method, contact areas between the grains bear intense mass transfer, which may be differentiated dependent on the pressure, current, voltage, heating rate, and exposure time. As a result, there is a high degree of scattering in the mechanical properties of these components, which have an effect on the cutting performance of the sintered WC cutting tool.

During the typical sintering process, the solid-state sintering mechanism is particularly important in the sintering behavior of pure tungsten carbide with finer grades [33]. Thus, a longer holding time is required so that diffusion and creep flow controlled mechanisms provide the final densification, but the prolongation of the holding time leads to significant, undesirable grain growth [34]. In contrast, electroconsolidation promotes the redistribution of the vacancies that contribute to the mass transfer, as is described in [35]. The equilibrium concentration of the vacancies near a pore is higher than that near the flat surface. As a result, between the core of the porous body and its surface, the gradient of vacancy concentration appears and forces them toward the surface. The reverse transition of the atoms contributes to the diffusional disappearance of the pores, unlike can be expected from Frenkel’s theory, where the effective self-diffusion coefficient is determined by the excessive thermal vacancies caused by the curvature of the pores. The vacancies transition takes place along the easiest diffusional track, and the velocity of their movement is increased according to the following row of order: volume–dislocation–boundary –inner surface–outer surface. Thus, a vacancy transition takes place across the areas of weakened chemical bonds, and the process is accumulative energy-consuming in the case of solid bodies. The concentration of the vacancies is determined by the activation mechanisms, which can be performed at the expense of either externally supplied energy or the internally accumulated energy. In addition, a certain role in mass transfer plays thermal diffusion, and temperature gradient has a favorable impact on the compaction of large pores. It should be noted, however, that the sintering mechanism depends directly on only two main phenomena, namely, the rapid increase of the contact surface between particles and the decrease of the distances between the centers of the particles. Other phenomena play only auxiliary roles in the electroconsolidation of WC nanopowders.

Since the sintering process pushes the entire system of separated particles toward the thermodynamical equilibrium, the excessive energy of the system is decreasing. Thus, this very energy is the motor of consolidation and it depends on the dimensions of the starting particles. Especially in the initial stages of electroconsolidation, the nano-dispersed particles exhibit higher resistance due to a large number of contact areas and related inter-particle contact resistances. The heat emission is linked to the inter-particle contacts, and thus, it increases when the particles are smaller.

In the hot pressing device with a directly applied current, no additional heat sources are needed. Thus, the consumed power is the product of the powder’s electrical resistance and the square of the amperage. The latter is not dependent solely on the resistance, hence, consumed power is not directly correlated with particle dimension.

**Figure 6:**
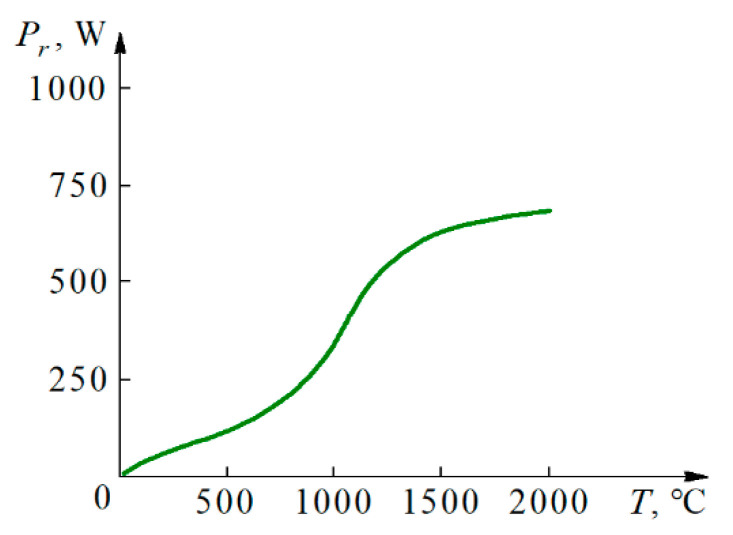
WC sintering power consumption *P_r_* versus temperature.

Figure 6 illustrates the functional relationship between the power on the secondary coil of the transformer *P_r_* and the sintering temperature. The *P_r_* is the product of electrical power and the respective number of alternating current cycles at a frequency of 50 Hz. Its value increases slowly up to ca. 1000 °C, then doubles at 1250 °C, and then again increases slowly up to 2000 °C.

**Figure 7:**
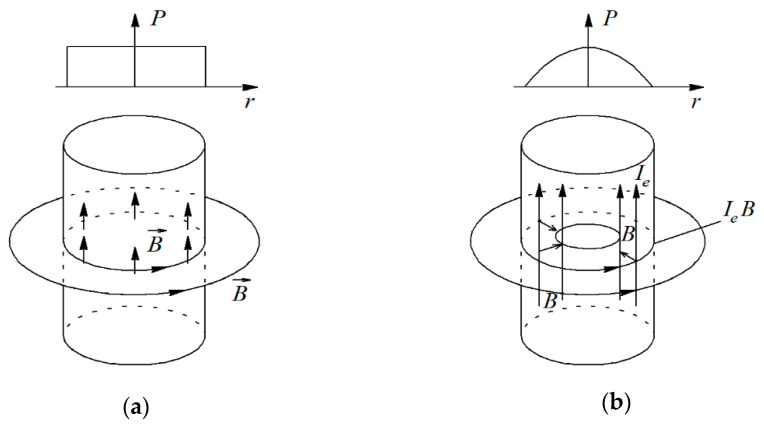
Pinch effect in the sintered powder volume: (**a**) Uniform current distribution along cross-sectional area; (**b**) Conductor with the surface current.

The pressure distribution during hot pressing can be assumed similar to that of metal powders, as it is described in [36]. It can be described as a pinch effect with uniform distribution of the electric current *I* in the cylindrical volume of the sintered powder with cross-sectional area *s*. The current density *I_e_ = I/s* is constant and induction B has azimuth direction, while the force *I_e_ ∙B* is directed radially, as it is shown in Figure 7.

**Figure 8:**
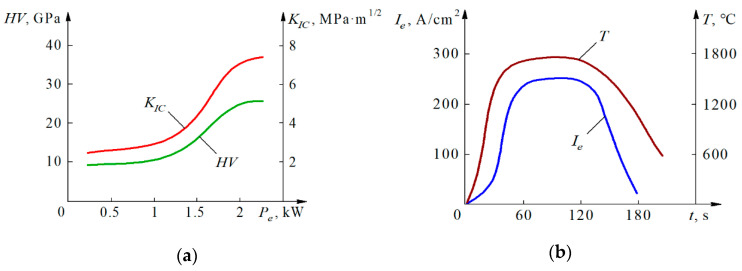
Effect of the process parameters on the characteristics of sintered WC: (**a**) Impact of the electric power *P_e_* on hardness *HV* and fracture toughness *K_IC_*; (**b**) Changes of the current density *I_e_* and temperature *T* during the process.

Under these assumptions, it can be calculated how the hardness and fracture toughness of as-sintered material is dependent on the electrical power *P_e_*. Figure 8 presents the graphs of mechanical properties of the sintered WC cutting inserts dependent on the power *P_e_* [kW] and changes of the current density and temperature during the process of electroconsolidation.

It should be noted that at powers below 1.2 kW, the increase of the hardness and fracture toughness is insignificant. In the interval between 1.2 and 2.0 kW, both *HV10* and *K_IC_* can be increased two times, but above 2.0 kW the improvement is very small. The temperature generally repeats the graph of current density *I_e_*, but decreases more slowly after 120 s.

**Figure 9:**
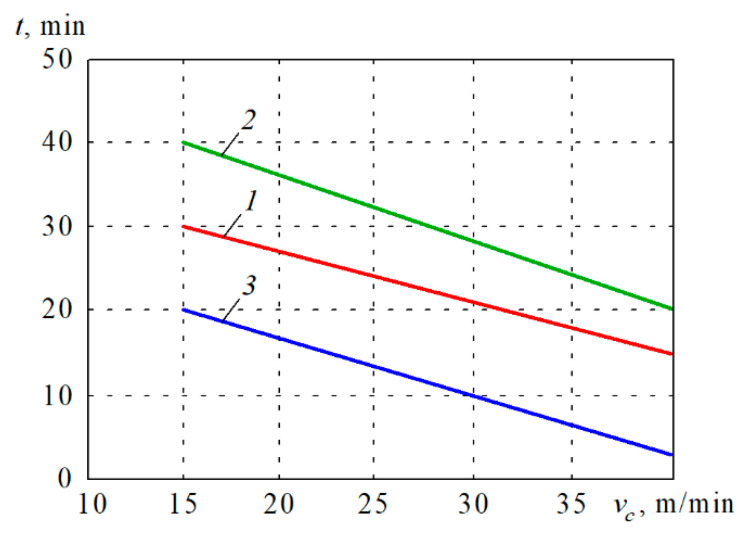
Durability of cutting inserts at different cutting speeds when machining TiC/Fe composite: 1—WolCar, 2—CBN, 3—BK8.

These findings contributed to the fabrication of highly wear-resistant cutting inserts out of binderless tungsten carbide nanopowders. Figure 9 compares the durability of WolCar inserts with WC-Co composite BK8, and cubic boron nitride (CBN) inserts. Wear criterion was the critical flank wear *VB* = 0.4 mm. From the graph, it can be seen that at the cutting speed *v_c_* = 15 m/min, WolCar, CBN, and BK8 inserts reached the critical flank wear in 30, 40, and 20 min, respectively.

When machining TiC/Fe composite, binderless WolCar performs 50% better compared to WC-Co cutting insert at low speeds, but the increase of the *v_c_* worsened the performance of BK8 to a higher degree than that of WolCar. In particular, when *v_c_* = 40 m/min, WolCar is able to work 70% longer than BK8. In this respect, WolCar cutting inserts performed more like CBN inserts.

**Figure 10:**
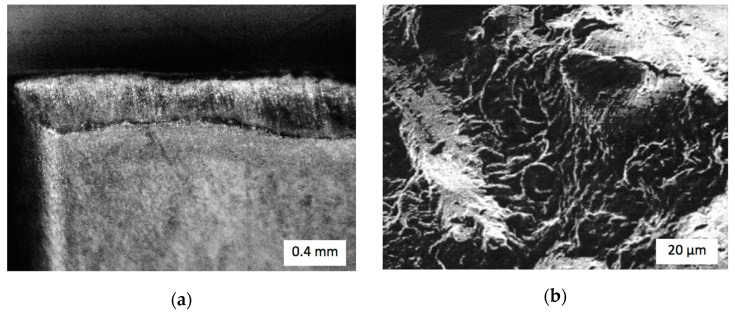
Flank wear of WolCar cutting inserts: (**a**) Photo; (**b**) SEM image.

At the speed range between 15 and 40 m/min, WolCar exhibited durability at the level of 75% of that performed by CBN cutting edges. An increase of speed from *v_c_* = 15 m/min up to *v_c_* = 40 m/min shortened the working time of both types of inserts by 50%. Due to the composite structure of TiC/Fe with ceramic inclusions in the iron matrix, the wear mechanism in the inserts was predominantly an abrasive one, as seen in Figure 10.

Neither larger cracks nor detachments were found in the WolCar worn surface layer. The presence of a slight built-up edge formation was noted after a higher cutting speed of *v_c_* = 40 m/min, which could have been eliminated with the application of a lubricant and cooling fluid. Thus, it was confirmed that the bulk structure of the sintered binderless WC retained its strength and fracture toughness under heavy loads during TiC/Fe machining.

## 5. Conclusions

From the investigations, the main conclusions can be driven as follows. First of all, it was possible to fabricate sintered cutting inserts out of pure WC nanopowder, without a cobalt binder. A dramatic reduction of the grain sizes contributed to the improvement of the mechanical properties as compared to the WC-Co composites. This feature made the binderless WC cutting inserts feasible for machining the TiC/Fe composite.

The most important finding is about the increased durability, especially at higher speeds. When machining 13CrMo4 steel at a cutting speed of *v_c_* = 100 m/min, the BK6 edges became worn out after 10–12 min, while the WolCar inserts were able to work as long as ca. 30 min.

In the case of the difficult-to-cut TiC/Fe composite, the superiority of binderless nanostructured nWC inserts is even more spectacular. Related to the cubic boron nitride (CBN) cutting inserts, at *v_c_* = 15 m/min, BK8 exhibited only 50% of its durability, while WolCar reached 75%. At a higher speed of *v_c_* = 40 m/min, the durability of the BK8 insert dropped down below 20%, while WolCar retained 75% of the CBN durability.

The abovementioned findings indicate an interesting and important direction of further research on machining of such materials as TiC/Fe composite or the high-speed cutting of steels. In particular, it is planned to perform tribological tests, as well as investigations to complete the wear curves. Another direction of further research is the measurement of cutting forces, dynamics of the cutting, and the resulting surface integrity of the machined parts. Moreover, it is necessary to work out optimized geometry of the cutting edges related to the specific machining tasks, to find out the most effective applications of this tool material, as well as to confirm experimentally its recyclability.

## Figures and Tables

**Table 1:** Chemical composition of the materials.

Material	Main Compounds
TiC/Fe composite	TiC (45%) and Fe (55%)
13CrMo4 [21]	C (0.8–0.18%), Si (max. 0.35%), Mn (0.4–1%), Cr (0.7–1.15%), Mo (0.4–0.6%), Cu (max. 0.3%) other (<0.1%), and Fe

**Table 2:** Characteristics of the WC samples sintered at different parameters from the submicron powders (samples #1–3), nanopowder (nWC), and cobalt binder (BK8).

WC Sintered Samples	Sintering Parameters		Relative Density, %	Grain Size, Μm	Hardness HV10, Gpa	Fracture Toughness *K_IC_*, Mpa∙M^1/2^
	Temperature, °C	Time, Min				
Sample #1	1630	1	98.7	0.5	24.3 ± 0.5	9.1 ± 0.5
Sample #2	1750	20	99.1	2.1	20.3 ± 0.5	8.2 ± 0.5
Sample #3	1800	20	99.8	5.5	18.4 ± 0.5	7.6 ± 0.5
WolCar (nWC)	1750	1	99.2	0.1	26.4 ± 0.5	8.5 ± 0.5
BK8	1450	60	99.0	3–4	16 ± 0.5	12 ± 0.5

## Data Availability

Data available on request due to privacy restrictions.
